# Multi-parametric functional ultrasound imaging of cerebral hemodynamics in a cardiopulmonary resuscitation model

**DOI:** 10.1038/s41598-018-34307-9

**Published:** 2018-11-06

**Authors:** Charlie Demené, David Maresca, Matthias Kohlhauer, Fanny Lidouren, Philippe Micheau, Bijan Ghaleh, Mathieu Pernot, Renaud Tissier, Mickaël Tanter

**Affiliations:** 1 Institut Langevin, ESPCI ParisTech, Paris Sciences & Lettres Research University, CNRS UMR7587, INSERM U979 Paris, France; 2 Inserm, U955, Equipe 03, Créteil, France; 3 UMR_S955, UPEC, Ecole Nationale Vétérinaire d’Alfort, 94700 Maisons-Alfort, France; 4 Mechanical Engineering Dpt, Université de Sherbrooke, Sherbrooke, QC Canada

## Abstract

Patient mortality at one year reaches 90% after out-of-hospital cardiac arrest and resuscitation. Temperature management is one of the main strategies proposed to improve patient outcome after resuscitation and preclinical studies have shown neuroprotective effects when hypothermia is achieved rapidly, although the underlying mechanisms have not yet been elucidated. State-of-the-art brain imaging technologies can bring new insights into the early cerebral events taking place post cardiac arrest and resuscitation. In this paper, we characterized cerebral hemodynamics in a post-cardiac arrest rabbit model using functional ultrasound imaging. Ultrasound datasets were processed to map the dynamic changes in cerebral blood flow and cerebral vascular resistivity with a 10 second repetition rate while animals underwent cardiac arrest and a cardiopulmonary resuscitation. We report that a severe transient hyperemia takes place in the brain within the first twenty minutes post resuscitation, emphasizing the need for fast post-cardiac arrest care. Furthermore, we observed that this early hyperemic event is not spatially homogeneous and that maximal cerebral hyperemia happens in the hippocampus. Finally, we show that rapid cooling induced by total liquid ventilation reduces early cerebral hyperemia, which could explain the improved neurological outcome reported in preclinical studies.

## Introduction

Ultrafast Doppler (UfD)^1^ is a breakthrough vascular imaging technology which is up to 40 times more sensitive than conventional ultrasound Doppler^2^. UfD can detect a wide range of blood flow velocities (from 10′s cm/s down to 1 mm/s) and provide the Doppler frequency spectrum analysis in all pixels of the acquired image. The development of UfD led to cerebral or cardiac blood flow characterization in humans^3,4^ and to functional ultrasound imaging of the brain through sequential UfD acquisitions used to map neurovascular coupling^5,6^. Besides neuronal dynamics, functional ultrasound imaging can play a role in the characterization of cerebral hemodynamic autoregulation.

In the field of neuroprotection, it is well-known that resumption of spontaneous circulation (ROSC) after a cardiac arrest is not sufficient for a complete recovery of the patient as post-anoxic lesions occur after resuscitation^7^. Target temperature management has been proposed to improve the neurological status and the survival rate after resuscitation. In humans, such management is recommended through the induction of hypothermia (33 °C)^8^ or sub-normothermia (36 °C)^9^. In animal studies, it has been shown that the cooling strategies that achieve very rapid hypothermia after resuscitation provide much greater benefits. One of those strategies is the total liquid ventilation (TLV) of the lungs with temperature-controlled perfluorocarbons, which was shown to rapidly cool the entire body in both small and large animals^10–14^. The benefits of such rapid hypothermic with TLV have been experimentally established both in terms of neuroprotection and sepsis mitigation after cardiac arrest in rabbits as compared to conventional cooling^10,11,13^. However, understanding the very early pathophysiologic events occurring after cardiac arrest remains critical to determine the ideal therapeutic window for hypothermia induction. Among them, early alterations of cerebral hemodynamics represent a major, potential therapeutic target.

Here we present a technical report on multi-parametric functional ultrasound imaging of cerebral hemodynamics during the first hour post-resuscitation in a cardiac arrest model. To show the usefulness of the technique, we imaged the early hyperemic and hemodynamic regulation events that occur in the brain with and without rapid hypothermic TLV.

## Methods

### Study design and animal handling

**Figure 1:**
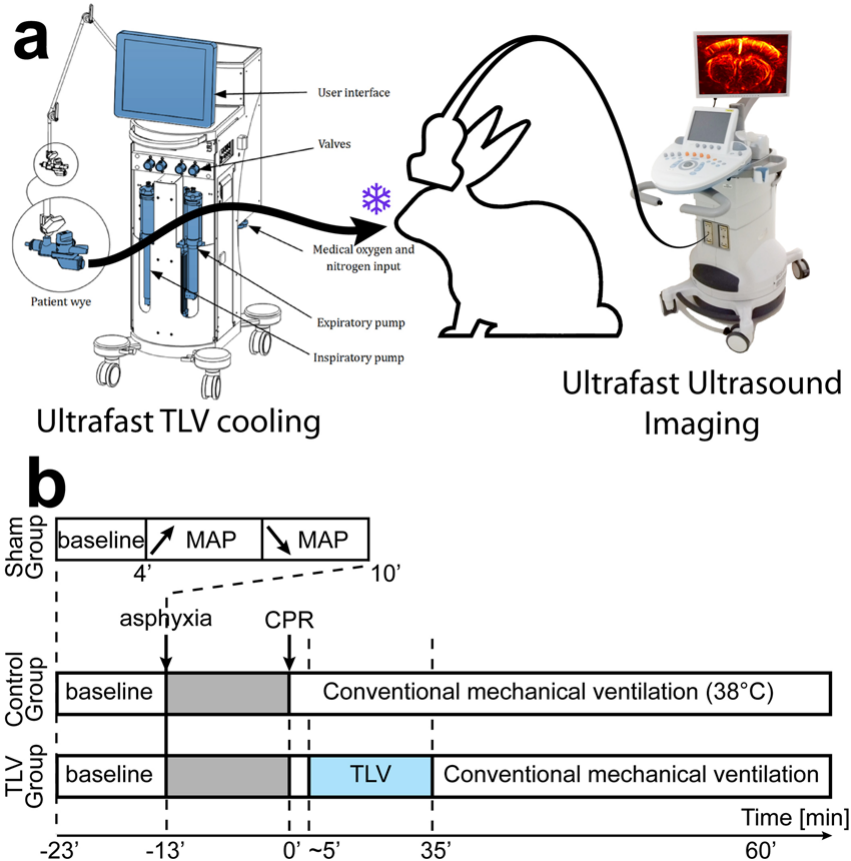
(**a**) Experimental setup schematics. Total liquid ventilation (TLV) is only used in the TLV group to induce whole body rapid cooling in rabbits during 30 minutes, lowering their body temperature down to 33 °C. Ultrafast Doppler imaging enables to monitor continuously brain hemodynamics. (**b**) Detailed time course of the experiment in the 3 groups: only 10 minutes for the Sham group to explore a certain range of MAP; and a total of 90 minutes both for Control and TLV groups subdivided as follows: 10 minutes baseline followed by 13 minutes asphyxia, cardiopulmonary resuscitation (<5 minutes), 30 minutes total liquid ventilation in the TLV group, conventional ventilation in the control group, and finally follow up for 55 minutes. The origin of time t = 0′ is chosen at the beginning of the CPR (23 minutes after the beginning of the experiment).

We conducted experiments in agreement with European regulations and the protocol was approved by local ethical committee (Comité d’Ethique enregistré auprès du Comité National de Réflexion Ethique sur l’Expérimentation Animale sous le n°16, AnSES (Agence nationale de sécurité sanitaire de l’alimentation, de l’environnement et du travail)/ENVA (Ecole nationale vétérinaire d’Alfort/UPEC (Université Paris est Créteil)). A cohort of 16 male New Zealand (NZ) rabbits was used. They were dispatched in 3 groups: a Sham group (N = 6), a Control group (N = 5) and a TLV group (N = 5). After deep anesthesia (zolazepam/tiletamine/pentobarbital, each 25 mg/kg i.v.), rabbits were intubated, received a neuromuscular blockade (cisatracurium besylate 0.2 mg/kg, i.v.), mechanically ventilated and subsequently a craniotomy was performed to obtain an acoustic window for brain imaging. Anesthesia was maintained by additional administration of pentobarbital. We kept the meninges intact. Mean arterial pressure (MAP) was measured using a catheter inserted in the femoral artery, and cerebral, oesophageal and rectal temperature were monitored. The head of the rabbit was maintained in a stereotaxic frame (David Kopf Instruments) in order to minimize movement during imaging. We mounted the ultrasound probe (6.4 MHz linear array, Vermon, France) via a mechanic arm onto the stereotaxic frame attached to the surgery table in order to maintain the desired coronal view throughout the imaging experiment (Fig. 1a). For the Sham group, the aim was to evaluate the variation of cerebral UfD signal over a wide range of MAPs in animals that did not encounter any cardiac arrest. The Sham rabbits’ brains were imaged at a rate of one image every 6 seconds over a baseline period of 4 minutes, followed by a 6 minutes period during which we slowly induced gradual MAP variations by compression of the abdominal aorta (Fig. 1b). Rabbits of the Control and TLV groups were continuously imaged at a rate of one image every 10 seconds over the course of the following procedure: first, a 10 minutes rest period meant to record hemodynamic baseline states in every animal. Respiratory arrest was induced by disconnecting mechanical ventilator for 13 minutes to induce subsequent cardiac arrest. Cardiac arrest typically occurred after 5 minutes of asphyxia. After 13 minutes of asphyxia, the animals were resuscitated by external chest compression combined to intravenous epinephrine administration (15 μg/kg) and resuming of conventional mechanical ventilation (time 0′). Animals in the Control group were handled at their physiological temperature throughout the experiment. Animals in the TLV group were connected to the liquid ventilator one minute after ROSC and for a total duration of 30 minutes (Fig. 1b). TLV lowered whole body temperature from 38 to 32 degrees within 15 minutes. After the 30 minutes of TLV, animals resumed conventional gas ventilation and temperature was maintained at 32 degrees. In both Control and TLV groups, arterial pressure was monitored during a one-hour follow-up period post resuscitation. Animals received continuous adrenaline infusion in order to maintain their MAP at 80 mmHg. To sum up, we continuously imaged cerebral hemodynamics over the 90 minutes of experimental protocol: image acquisitions contain a 10 minutes baseline period (from −23′ to −13′), followed by a 13 minutes respiratory arrest period (from −13′ to 0′), and a 67 minutes post arrest follow-up period (from 0′ to 67′). All animals were terminated according to European guidelines immediately after the experiment.

### Ultrasound imaging pulse sequence

**Figure 2:**
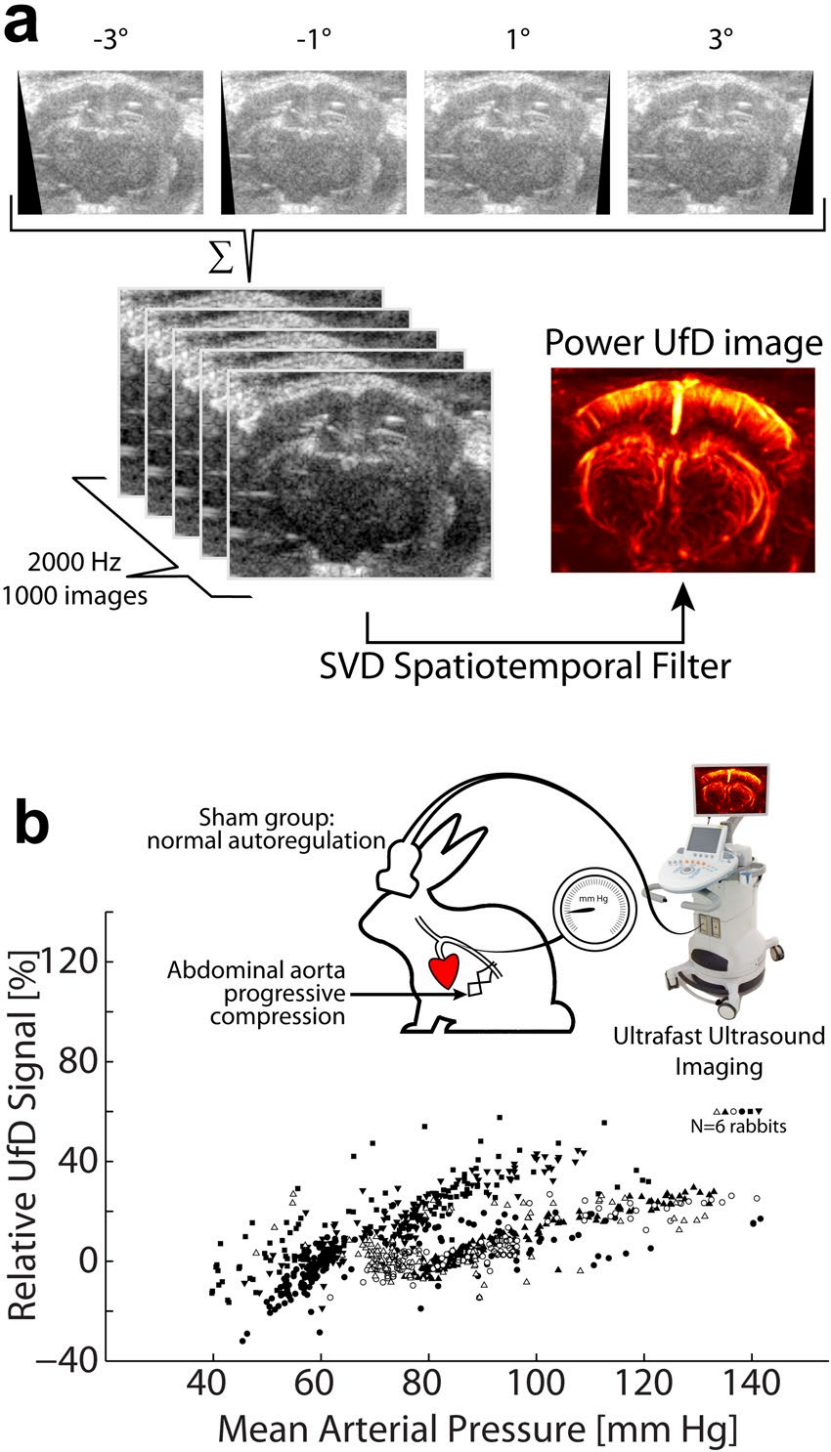
(**a**) Ultrafast Doppler pipeline acquisition: four ultrasonic images are obtained by sending tilted ultrasound plane waves in the medium. Those images are coherently compounded to increase contrast and resolution. This process is repeated 1000 times at a framerate of 2000 Hz. Each stack of 1000 images is filtered to retrieve blood signal. (**b**) Cerebral autoregulation in normal rabbits (Sham group, N = 6) assessed via the acquisition of relative UfD as a function of mean arterial pressure. Arterial blood pressure was increased by progressive compression of the abdominal aorta. When the mean arterial blood pressure is modified by an external event, individuals tend to regulate their cerebral blood flow to a constant value. On N = 6 rabbits (each one being depicted by different symbols) undergoing rather important modifications of their mean arterial blood pressure (up to 140 mmHg), the relative UfD signal show a moderate increase (lower than 60%).

We acquired UfD images in a central coronal slice of the brain (capturing the cortex, the hippocampus, and the thalamus) at a frame rate of 2000 Hz (Fig. 2a), enabling quantitative evaluation of the cerebral blood volume and resistivity changes in the insonified blood vessels. The ultrasound brain imaging sequence was programmed to enable high quality ultrafast Doppler image acquisition every 10 seconds during 90 minutes. The sequence relied on the coherent compounding of 4 tilted plane wave emissions (3 cycle pulse, angled at −3°, −1°, 1°, 3°) in brain section of interest and fired at a 8 kHz ultrasound pulse repetition frequency, which leads to a 2 kHz sampling frequency after coherent compounding^15^ (Fig. 2a). Considering the 6.4 MHz transducer imaging frequency, this allowed to sample cerebral blood flows inferior to 24 cm/s^16^, which is enough for rabbit brain circulation. Each ultrafast Doppler acquisition (0.5 s, 1000 images) covered 2 cardiac cycles in NZ rabbits under normal physiological conditions.

### Computation of relative cerebral blood volume and resistivity maps

**Figure 3:**
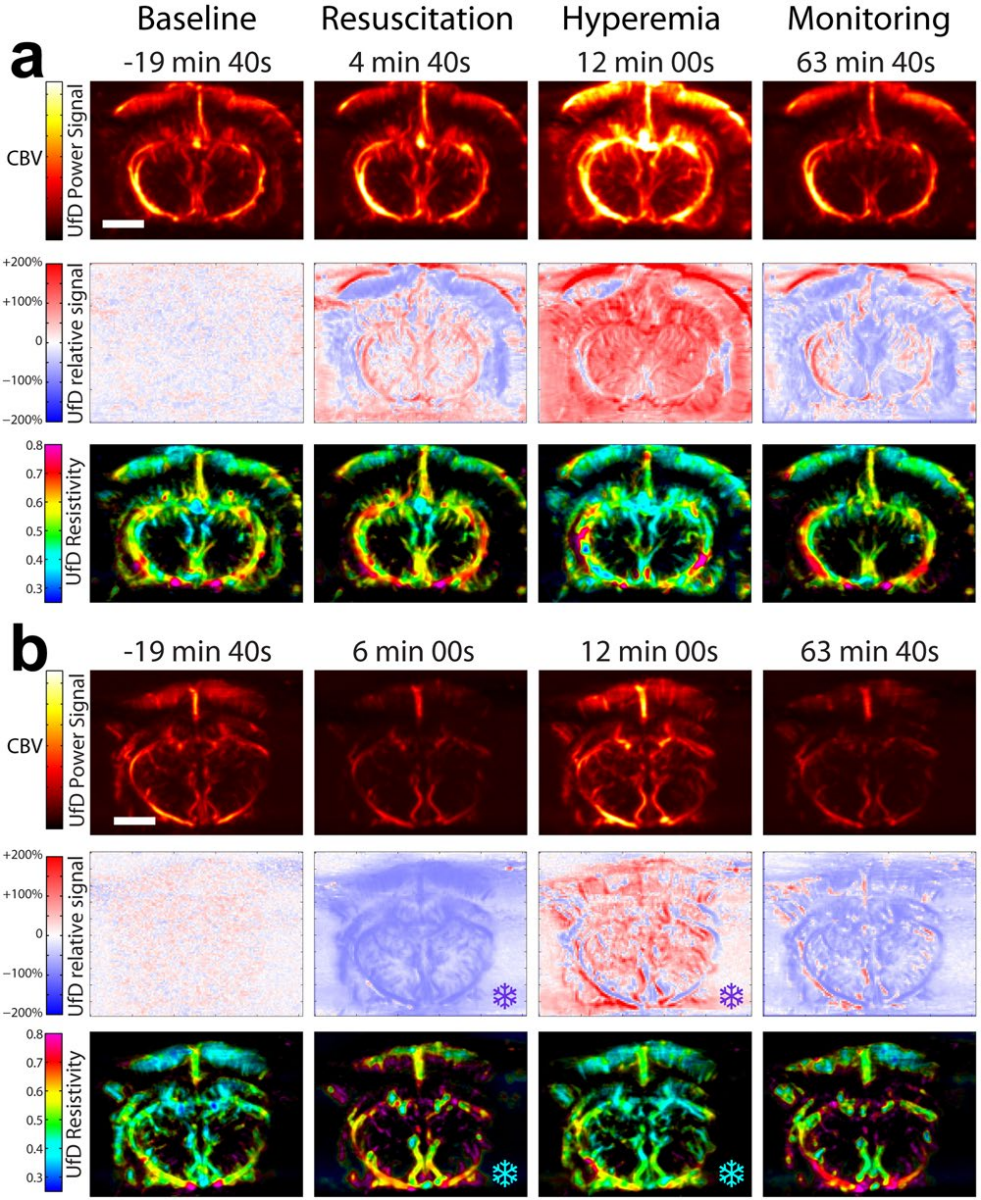
Multiparametric imaging of the brain derived from ultrafast Doppler acquisitions. UfD Power signals (1^st^ row) display intensities proportional to cerebral blood volume. UfD relative signals (2^nd^ row) provide the percentage of increase or decrease in cerebral blood volume with respect to baseline level. UfD resitivity maps (3^rd^ row) the ratio of the range of arterial velocity during the cardiac cycle to the arterial systolic peak velocity. The first column (t = −19 min 40 s) presents baseline state, the second column (t = ~4–6 min) presents the brain status immediately after resuscitation, the third column (t = 12 min) reports brain condition at maximal hyperemia, and the last column (t = 63 min 40 s) presents brain condition one hour after CPR. (**a**) Control group. (**b**) TLV group.

In our experimental datasets, we filtered out tissue clutter from blood flow signal using a spatiotemporal singular value decomposition filter^17^. We then processed the filtered data by computing the signal energy in each pixel to build “Power Doppler” maps of brain vasculature in which intensity is proportional to cerebral blood volume^18^. We processed the data further to display in each pixel relative blood volume variations over time with respect to baseline level (second rows of Fig. 3a,b). Finally, we performed a time-frequency (spectrogram) analysis of the filtered data in each pixel in order to calculate the mean blood flow speed as the first statistical moment of the spectrum of each time point. By analyzing the evolution of this blood flow speed during the cardiac cycle we could derive resistivity maps of brain vasculature^4^, resistivity being defined as the ratio between the range of blood flow speed and the maximum blood flow speed (third rows of Fig. 3a,b). Strong differences between systolic (maximum) and diastolic (minimum) blood flow velocities lead to high resistivity values.

### Regional analysis of early hyperemia and its mitigation by TLV

**Figure 4:**
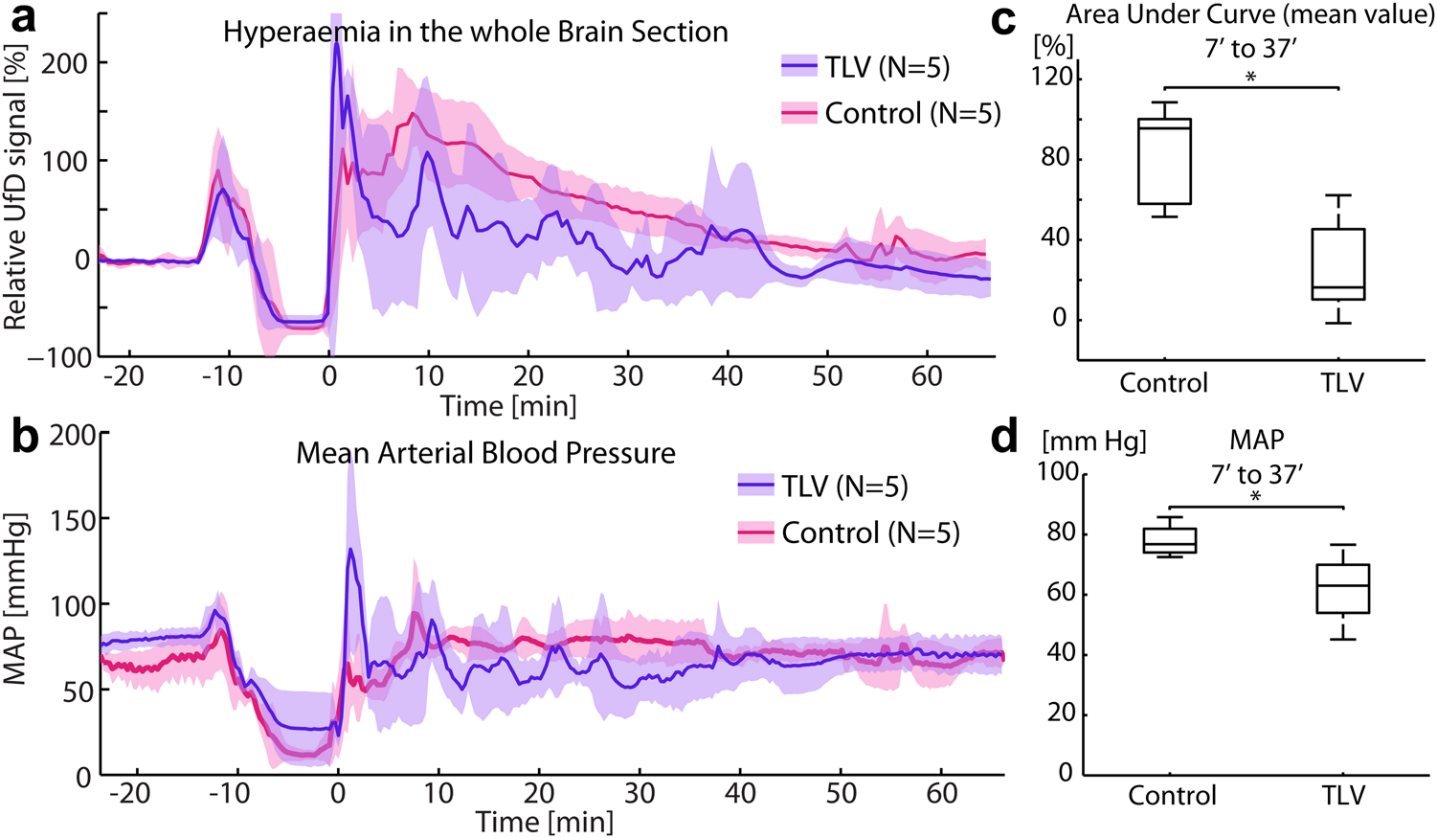
Time course of the brain average Ultrafast Doppler signal in the 2 groups, compared with the time course of the mean arterial blood pressure (MAP). (**a**) UfD level (proportional to the blood volume) in Total Liquid Ventilation (TLV) group (blue) compared to Control group (red) over the course of the experiment (baseline, asphyxia, CPR and monitoring post resuscitation). Solid line: average value on N = 5 individuals, overlay: corrected sample standard deviation. (**b**) Mean arterial blood pressure for both groups during the course of the experiment. Solid line: average value, light color: corrected sample standard deviation. (**c**) Area under the curve of UfD signals for both groups between t = 7 minutes and t = 37 minutes. Hyperemia was significantly higher in the control group than in the TLV group (p < 0.05, Mann Whitney U test). Box plots show the median and interquartile and extreme AUC values per group. (**d**) Average arterial blood pressure during the same time period (t = 7 minutes to t = 37 minutes). The MAP was also significantly different between the two groups (p < 0.05, Mann Whitney U test) with a slightly lower tendency for the TLV group, with average values in the physiological range.

**Figure 5:**
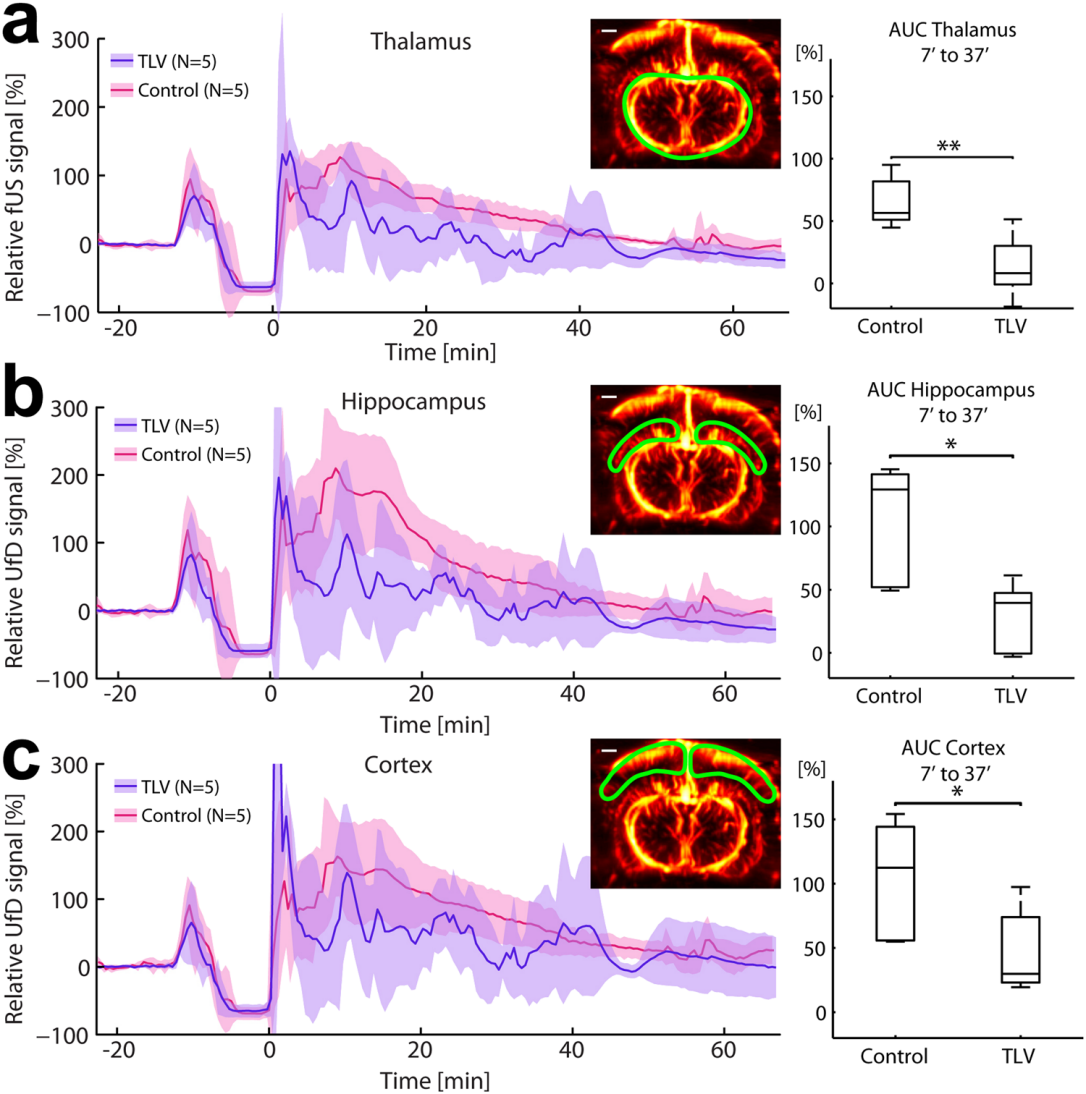
Regional CBV disparities during hyperemia. Box plots show the median, interquartile and extreme AUC values per group. (**a**) In the thalamus, hyperemia was moderate (up to +125%) in the Control group. The difference was statistically significant compared to the TLV group (*p* = 0.008). (**b**) Hyperemia in the hippocampus. UfD intensities reached an absolute maximum of +200% compared to the baseline level, which is significantly (*p* = 0.016) higher than for the TLV group (maximum of +100% for a very short period; median normalized AUC = +40%). (**c**) In the cortex the difference between the control group and the cooled subjects was less visible but still statistically significant (*p* = 0.048).

We averaged spatially in regions of interest the power UfD signals as a function of time in each group of 5 rabbits (Control and TLV groups) in order to depict statistically significant cerebral hemodynamic variations over the course of the experiment. Once spatially averaged, UfD signals were normalized to their baseline value. In Fig. 4, we report the mean relative UfD signal as a function of time computed over the entire brain slice, whereas Fig. 5 presents regional relative UfD variations — *i.e*. the analysis of UfD signals over time in three distinct brain regions: the cortex, the hippocampus and the thalamus. This was achieved by manually segmenting the thalamus, cortex and hippocampus in our Power UfD vascular maps and averaging UfD signals over those selected areas (see Fig. 5a). We assessed the magnitude of the hyperemia event by computing the area under the curve (AUC) of the mean UfD curves between t = 7 and t = 37 minutes (time span of TLV, the cardiopulmonary resuscitation and set up of the TLV took 2 to 7 minutes depending on the individual, as such we set 7′ as the starting point of the post resuscitation period). Finally, we compared the statistical significance of the hyperemia event in the control group to the mitigated hyperemia event in the TLV group using a paired Mann-Whitney U test. We report these results in a box plot showing the median and interquartile and extreme AUC values per group (Control versus TLV) in Figs 4 and 5. To compare effect size between groups in different regions (cortex vs thalamus vs hippocampus), AUC was compared during the peak of the hyperemia (AUC between 7 and 22 minutes), and effect size was measured using a Cohen’s *d* estimation for each cerebral region, and validated using a one-tailed Mann-Whitney U test. As a reminder, the Cohen’s *d* value measures how much means are differents among groups. A d value > 0.8 is generally considered as large^19^.

### Principal component analysis of cerebral blood volume vs. mean arterial pressure

**Figure 6:**
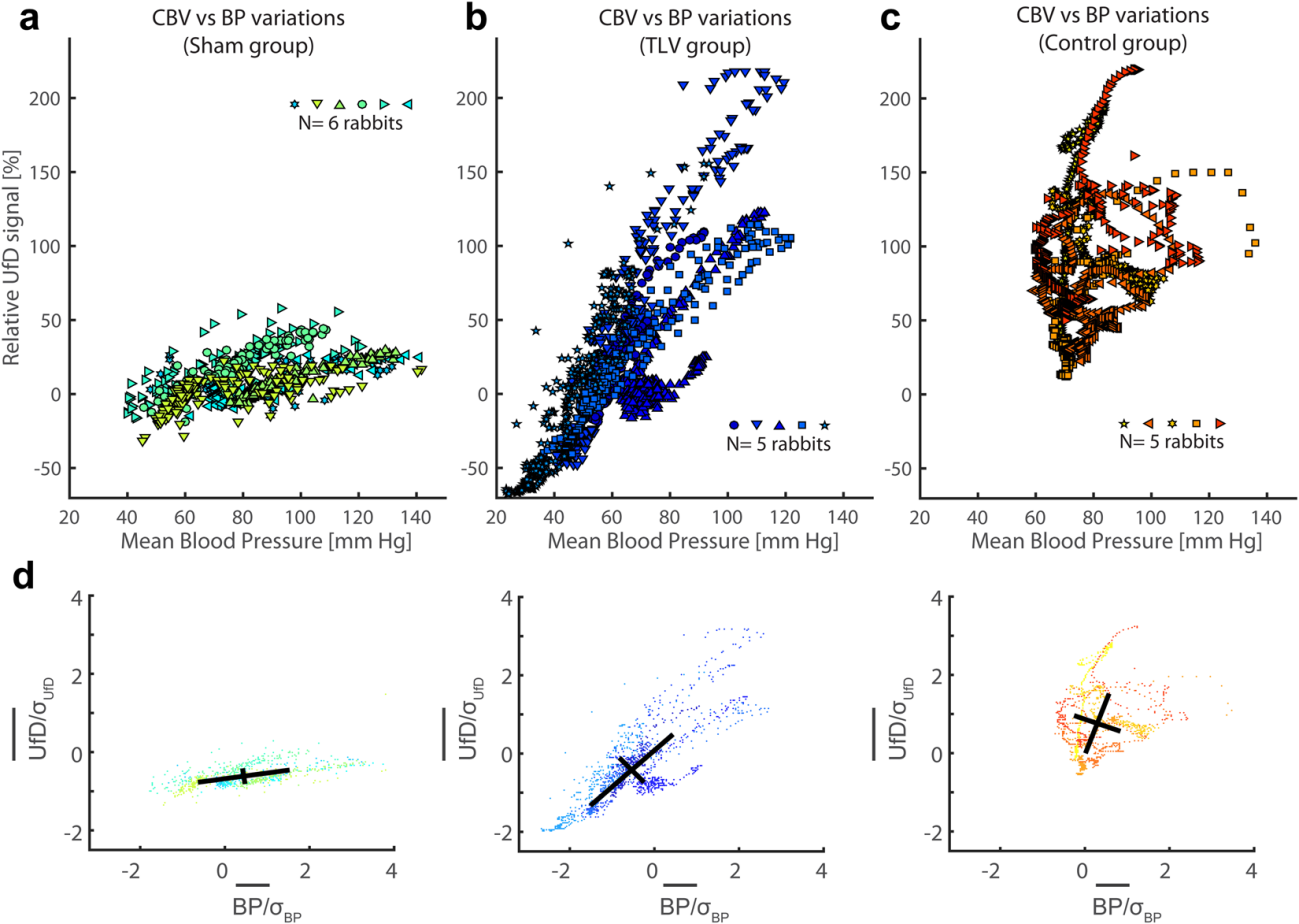
Cerebral autoregulation assessed on a UfD vs MAP graph, by comparing the effect of mean arterial pressure variation on the brain average UfD signal. (**a**) Autoregulation exhibit a plateau (almost flat slope) for the sham (normal rabbits) group. (**b**) Autoregulation is moderately altered, exhibiting a steep slope in the case of cardiac arrest followed by TLV treatment. (**c**) Autoregulation appears highly disturbed in the control rabbits post resuscitation, without real linear correlation anymore between UfD variations and MAP. (**d**) Representation of the standardized data, with the two principal component vectors represented.

As UfD intensities are proportional to cerebral blood volume (CBV)^20^, we used UfD intensity variations to map blood volume variations in the vasculature of the brain. We analyzed average cerebral blood volume (CBV) versus MAP variations for the three groups (Sham, Control and TLV). First, we mapped CBV as a function of MAP over the 10 minutes of the Sham imaging session (Fig. 6a). Second, we mapped post cardiac-arrest CBV versus MAP variations between 7′ and 37′ - i.e. the first 30 minutes after the resuscitation - for animals of the Control and TLV groups (Fig. 6b,c respectively). Figure 6d was generated by normalizing each variable as standards scores (we removed the mean value across all data and normalized by the standard deviation across all CBV and MAP data) and computing the principal component analysis for each animal group (baseline group, control group and TLV group). The complete set of vectors and associated weights obtained from these principal component analyses were (vi is the ith principal vector and λi the associated weight) Sham: v1 = (0.99, 0.14), λ1 = 1.20, v2 = (−0.14, 0.99), λ2 = 0.040; TLV: v1 = (0.73, 0.68), λ1 = 1.79, v2 = (−0.68, 0.73), λ2 = 0.18; Controls: v1 = (0.34, 0.94), λ1 = 0.66, v2 = (0.94, −0.34), λ2 = 0.34.

## Results

UfD imaging was successfully used to generate high quality vascular maps of the rabbit brain vasculature (Fig. 2a). Blood vessels of the cortex, thalamus and hippocampus were clearly depicted. Figure 2b provides a reference to apprehend three important aspects for the study: (1) what is the physiological range for rabbit MAP?, (2) what is the corresponding UfD variation range, and (3) what characterizes cerebral autoregulation in sham animals? For rabbits, the literature reports an arterial systolic pressure of typically 90–130 mm Hg, a diastolic pressure of 80–90 mm Hg and a mean arterial pressure 80–105 mm Hg^21,22^. Those values can be slightly lowered by the anesthesia. The MAP range explored in Fig. 2b covers those typical values to a large extent, including the hypo- and hypertension cases. In addition, it is important to note that physiological UfD variations values reported in the brain typically range from 10 to 50% when neurons are implicated in a cognitive task triggering neurovascular coupling^5,23,24^. In normal rabbits (Sham group), the analysis of MAP values and relative cerebral UfD values revealed that autoregulation mechanisms maintains CBV variations in this physiological range over a wide range of MAP changes (Fig. 2b). This is expected as autoregulation is generally described as a plateau in the curve representing the evolution of cerebral blood flow as a function of arterial pressure^25^. This plateau is preceded by a positive slope characteristic of arterial collapse (the arterial pressure is too low to maintain the arteries opened), and followed by another positive slope corresponding to the “force-mediated” dilation of the vessels (the pressure is too high and dilate the vessels). On Fig. 2b we observe a plateau with a slight positive slope. In the 70 to 105 mmHg range, relative UfD variations averaged across the whole brain section did not exceed +45% (the few points above that threshold corresponding to very transient events) thanks to cerebral autoregulation. In the overall MAP range explored, the variation never exceeded 60% in all the studied individuals of the Sham group (N = 6). It should be noted that for the lowest values of MAP (40 to 60 mm Hg), the downward slope is steeper, suggesting that in this case, the rabbits enter into an arterial collapse regime previously described in the literature. This moderate variation of relative UfD for normal physiological levels is important for further understanding of unusual CBV variations which occur in subsequent cardiac arrest and resuscitation experiments.

In the Control and TLV groups, UfD monitoring captured three successive phases: (1) the baseline phase (hemodynamics at rest), (2) the cardiac arrest phase (induction of asphyxial cardiac arrest under deep anesthesia, with subsequent cadiopulmonary resuscitation, and (3) the post-cardiac-arrest phase (hemodynamic recovery after resuscitation). During the 10 minutes baseline period (Fig. 1b), the MAP measurement exhibited small fluctuations and stayed between 60 and 80 mm Hg, while relative UfD signal stayed around 0% (i.e. no increase or decrease) (Fig. 3a,b at t = −19 min 40 s). During the cardiac arrest phase, the physiological responses observed consisted of first a transient and compensatory MAP and heart rate augmentation, followed by a drop in MAP until cardiac arrest. Since UfD intensities are proportional to CBV, we used UfD intensity variations to map CBV variations in the vasculature of the brain for the entirety of the experiment (Fig. 3a,b, first rows). To further quantify hemodynamic variations, UfD values were normalized towards average baseline level, leading to parametric maps of relative UfD levels (Fig. 3a,b, second rows). Results are presented at four distinct times and cover all phases of the experiments. At baseline (t = −19 min 40 s), the relative blood volume variation is homogeneously low and the image appears mostly white. After resuscitation (t = 4 min 40 s for the individual in the Control group and t = 6 min for the individual in the TLV group), the parametric UfD maps showed diverse relative CBV maps among individuals. Such variations among individuals seem plausible since all rabbits do not react in the same way to cardiac massage and epinephrine injections. At t = 12 min, animals in the Control group showed a massive hyperemia occurring in the whole brain (Fig. 3a, second row), whereas for rabbits in the TLV group, the hyperemia event appeared less severe (Fig. 3b, second row, 12 min). In the Control group, we observed that the hippocampus endured maximal vasodilatation during hyperemic event. One hour after cardiopulmonary resuscitation (CPR) (t = 63 min 40 s), the relative UfD level was more or less the same in the two groups, and slightly lower than the normal physiological state level (at baseline), as described in previous studies^26–28^.

A second type of parametric imaging, referred to as resistivity index mapping^4^, was derived from UfD acquisitions and provides a potential explanation for the hyperemic event that relates to vascular tone. In the Control group, we observed that the resistivity of the cerebral vasculature was lower at the peak of hyperemia (Fig. 3a, 3^rd^ row, t = 12 min) than at baseline (Fig. 3a, 3^rd^ row, t = −19 min 40 s), whereas in the TLV group the resistivity values remained approximately the same at 12 min and −19 min 40 s (Fig. 3b, 3^rd^ row, 12 min and −19 min 40 s).

From a quantitative point of view, the hyperemia induced after CPR in the control group was beyond normal physiological limits - the whole brain UfD relative levels reached an average +150% ± 34% at peak as compared to baseline (Fig. 4a), indicating that the cerebral blood volume was 2.5 times higher than physiological level at baseline. Note that UfD variations between 23 and 26 minutes are due to strong motion artefacts induced by chest compression, which explains the large apparent dispersion in UfD values in that 3 minutes window. We computed the AUC over the 30 minutes that followed CPR and showed that TLV mitigates hyperemia as the average hyperemia event was significantly lower (*p* = 0.032) in the TLV group (mean +26% ±SD 25%) than in the Control group (mean 82.6% ±SD 26%). UfD imaging successfully provided insights into the dynamics of this hyperemia event which occurs very rapidly, since the maximal UfD level was reached on average only 10 minutes after CPR. It is critical to note that MAP remained in the normal physiological range (60 to 90 mmHg) for both groups of rabbits (TLV and Control) during the post cardiac arrest phase. Even if they exhibited a slight difference (considered significant, *p* < 0.05) (Control group: median 77 mm Hg, mean 78 ±SD5.6 mm Hg; versus TLV group: median 63 mm Hg, mean 62 ±SD13 mm Hg) (Fig. 4c,d), this difference cannot explain the tremendous CBV variations after CPR in the Control group. In comparison, at this physiological levels of pressure (between 80 et 90 mmHg), the average recorded relative UfD variation in normal rabbits (Sham group, Fig. 2b) was 13% ± 3%. These results suggest that hyperemia is probably related to an impairment of cerebral autoregulation – a hypothesis which is tested later in the paper.

UfD imaging also revealed that hyperemia in the Control group was not evenly distributed across the brain and allowed for the quantification of those regional variations (Fig. 5). The cerebral structure that endured the highest CBV increase in our imaging plane was the hippocampus (Fig. 5a). Relative UfD intensities reached up to +210% ± 74%, with an average value between 30′ et 60′ of +103% (mean +103% ±SD 48.1%) for the Control group compared to +28% (mean +28.2% ±SD 28.3%) for the TLV group. In the thalamus, hyperemia was also detected but its amplitude was lower than in the hippocampus: the peak value was +126.6% ±SD 19.3% and the average value between 30′ and 60′ of +65.4% ±SD 20.5% for the Control group compared with +13.6% ± SD 13.6% for the TLV group (Fig. 5b). The cerebral cortex exhibited the smallest UfD difference between the two groups (Fig. 5c), with an average value between 30′ et 60′ of +104% (median +112.4%, mean +104% ± std 46.5%) for the Control group compared to +47.4% (median 29.9%, mean +47.4% ± std 33.5%) for the TLV group. To support this statement on the strength of hyperemia among cerebral areas, the Cohen’s *d* effect size was calculated for each area (thalamus, hippocampus, cortex) between TLV and Control groups during the 15 minutes in which hyperemia was maximal (i.e. AUC was now calculated from 7′ to 22′). Hippocampus showed indeed the maximal effect (largest *d*) (*d* = 1.76, *p* = 0.016), followed by the thalamus (*d* = 1.081, *p* = 0.047) and finally the cortex (*d* = 1.08, *p* = 0.048). To conclude, UfD imaging highlighted significant regional hyperemia differences among cerebral structures in the Control group, and showed that hyperemia was mitigated by TLV in all structures, underlining the therapeutic efficiency of this rapid cooling strategy.

In order to test whether cerebral autoregulation is impaired by asphyxial cardiac arrest, we analyzed CBV variation as a function of MAP for the three animal conditions (Fig. 6). Cerebral autoregulation is usually described^25^ as a plateau of CBV across a wide range of physiological MAP variations. As expected, the Sham group (no cardiac arrest) exhibited a relatively flat CBV vs MAP scatter plot (Fig. 6a). For further quantification, we performed a principal component analysis (Fig. 6d) on reduced variables (i.e. variables normalized toward their standard deviation) from the datasets from Fig. 6a–c. In the new coordinate system, variance of the Sham group data is mainly explained by the first vector of the PCA: we obtained a weight ratio λ1/λ2 equal to 30 and the coordinates for the first vector v1 equal to (0.99, 0.14), i. e. a quasi-horizontal vector. This demonstrated that autoregulation is indeed a plateau in the Sham group. In the post-cardiac arrest groups, this plateau disappeared for both Control and TLV rabbits (Fig. 6b,c). However, in the TLV group (Fig. 6b), a linear relationship remained as indicated by the fact that the variance of the data is to a large extent explained by the first vector of the PCA (λ1/λ2 = 9.94). The slope was nonetheless considerably steeper (v1 = (0.73, 0.68)) than in the baseline group. In the control group (Fig. 6c), autoregulation appeared to be impaired as no linear trend could be derived from the variance of the CBV vs MAP plot (λ1/λ2 = 1.94, and v1 = (0.34, 0.94) and v2 = (0.94, −0.34)). These results tend to indicate that cerebral autoregulation mechanisms were no longer present post cardiac-arrest in the absence of rapid cooling of the brain, as there is no longer a clear correlation between MAP values and CBV values in the control group.

## Discussion

The first finding of this study is that UfD imaging is well-suited to unveil cerebrovascular function in intensive care models. In our animal model, it revealed an early hyperemia occurring within 30 minutes after ROSC and progressively returning to baseline values after 30 minutes. Our findings concur with post-resuscitation hyperemia observations reported in an earlier MRI study in rats^26^ (with a temporal resolution of 5 minutes). This observation emphasizes the need both for rapid imaging techniques versatile enough to be used in an operating room, and for rapid therapeutic strategies to counterbalance this non-physiological hemodynamic state, therefore supporting ultrafast cooling strategies such as TLV. The origin of this early hyperemic event is debated, and the trend and temporal dynamics differ from one animal model to the other^27–29^. In particular, one of the arguments put forward is adrenaline injection and their influence on blood pressure. Our results show that blood pressure variations cannot explain this early hyperemia.

A second finding is that TLV drastically reduces the magnitude of cerebral hyperemia, even in the first 15 minutes. Note that the long-term neuroprotective effect of ultra-fast cooling and TLV is already well-established^7,8,30^. Previous research by our group^13,31^ has shown a much better recovery in animals treated with TLV, and that damages caused to the blood brain barrier in the control group occurred in the first 2 hours following resuscitation^10^. This suggests that the early hyperemia reported in this paper may play a role in subsequent neurological damages and sepsis-like shock observed in the absence of ultrafast therapeutic hypothermia. Vascular resistivity mapping revealed measurable drops in arterial resistance after cardiac arrest in the control group, potentially suggesting that cerebral blood-flow autoregulation mechanisms are altered and that the tone of cerebral blood vessels is altered by cardiac arrest. Moreover, UfD imaging revealed significant regional disparities during hyperemia, showing in particular that the hippocampus was exposed to the highest effect size, when considering differences in the blood volume variation with and without cooling. These insights gained through UfD imaging of the brain concur with previous data that showed spatial disparities in cortex, hippocampus and thalamus in terms of microanatomy and blood brain barrier permeabilisation^10^.

An additional contribution of the present study is the proposed method to evaluate cerebral autoregulation efficiency. The CBV vs MAP scatter plots give hints on the severity of cerebral autoregulation impairments occurring for the Control and the TLV groups. The large hyperemic events were associated with the loss of a linear trend between CBV vs MAP in the control group, even though MAP was maintained within normal physiological limits after resuscitation, whereas animals of the TLV group retained a linear relationship (but with a very steep slope) between CBV and MAP.

In the present study, we decided to focus on large cerebral functional areas (cortex, hippocampus, thalamus) to maximally reduce possible positioning variations of the selected coronal imaging plane and therefore extract representative CBV variations for each structure. Positioning variations are due to the inter-individual brain shape variations and the complexity of the procedure, including a craniotomy of the rabbit prior to the experiment and positioning of the rabbit on the side for compatibility with the CPR. In the future, 3D Ultrafast Imaging will solve this problem by enabling the segmentation of complete cerebral substructures^32^.

A close look at Fig. 3 reveals disparities in the amplitude of hyperemia as a function of depth in the cortex, which raises questions about the implication of pia and meningia vessels in the observed hyperemia rather than penetrating vessels. To elucidate these variations, we conducted a 3-layer analysis of the cortical hyperemia in the cortex (Supplemental Fig. 1). It shows that for the control group, hyperemia is maximal in the first layer with relative UfD values up to +400% above baseline level. Note that even though such a layer analysis was possible, UfD imaging is a technology sensitive to axial blood flow velocities between 2 mm/s and 240 mm/s; this implies that we capture signals from arteries, veins, arterioles and venules, but not from capillaries using the combination of parameters implemented in this study. Moreover, in the 2D coronal section that was considered, most of the signal arises from penetrating arterioles and not from surface vessels (Doppler imaging is less sensitive to horizontally oriented vessels). Pia and meningeal vessels are restricted to a very thin layer (of 0.5 mm)^33^ which corresponds to only 2 pixels in depth in our images. The observed disparities for the measured hyperemia in the different layers of the cortex are most likely due to the fact that vessel calibers decrease with depth, and that our imaging method lacks sensitivity at the capillary level - rather than a separate behaviour of pia vessels compared to penetrating cortical vessels.

Finally, our study supports the need for new strategies which can induce therapeutic hypothermia immediately after cardiac arrest. Indeed, the therapeutic window appears to be very narrow for the mitigation of early hyperemia, which lasts about 30 minutes after cardiac arrest in our study. This might explain why delayed hypothermia, as currently induced in clinical trials, provides only marginal benefits^9^, when compared with the huge benefits of rapid hypothermia in laboratory studies. In the latter, rodents or rabbits can be rapidly cooled within minutes, whereas patients need at least several hours to reach comparable target temperatures. In this context, TLV could be a potent strategy for increasing cooling rates as conveyed by the benefits of rapid cooling highlighted in our study.

## Electronic supplementary material

Supplementary information
