# Supplementary material for: Multi-parametric functional ultrasound imaging of cerebral hemodynamics in a cardiopulmonary resuscitation model
Source: Sci Rep. 2018 Nov 6;8:16436. doi: 10.1038/s41598-018-34307-9 (PMC6219610; doi:10.1038/s41598-018-34307-9)
Supplement: Supplementary file 1 — Supplementary information [file 41598_2018_34307_MOESM1_ESM.docx]

Multiparametric functional ultrasound imaging of cerebral hemodynamics in a cardiopulmonary resuscitation model

Charlie Demené^1*^, David Maresca^1*^, Matthias Kohlhauer^2,3^, Fanny Lidouren^2,3^, Philippe Micheau^4^, Bijan Ghaleh^2,3^, Mathieu Pernot^1^, Renaud Tissier^2,3†^, Mickaël Tanter^1†^.

1. Institut Langevin, ESPCI ParisTech, Paris Sciences & Lettres Research University, CNRS UMR7587, INSERM U979, Paris, France

2. Inserm, U955, Equipe 03, Créteil, France.

3. UMR_S955, UPEC, Ecole Nationale Vétérinaire d’Alfort, 94700, Maisons-Alfort, France

4. Mechanical Engineering Dpt, Université de Sherbrooke, Sherbrooke, QC, Canada

* Charlie Demené & David Maresca contributed equally to this work.

† *Renaud Tissier & Mickaël Tanter equally co-supervised this work.*

**Corresponding author**: Charlie Demené, charlie.demene@espci.fr

Postal address: Institut Langevin

17 Rue Moreau

75012 Paris, FRANCE

Telephone: +33 80 96 33 83

Supplementary information


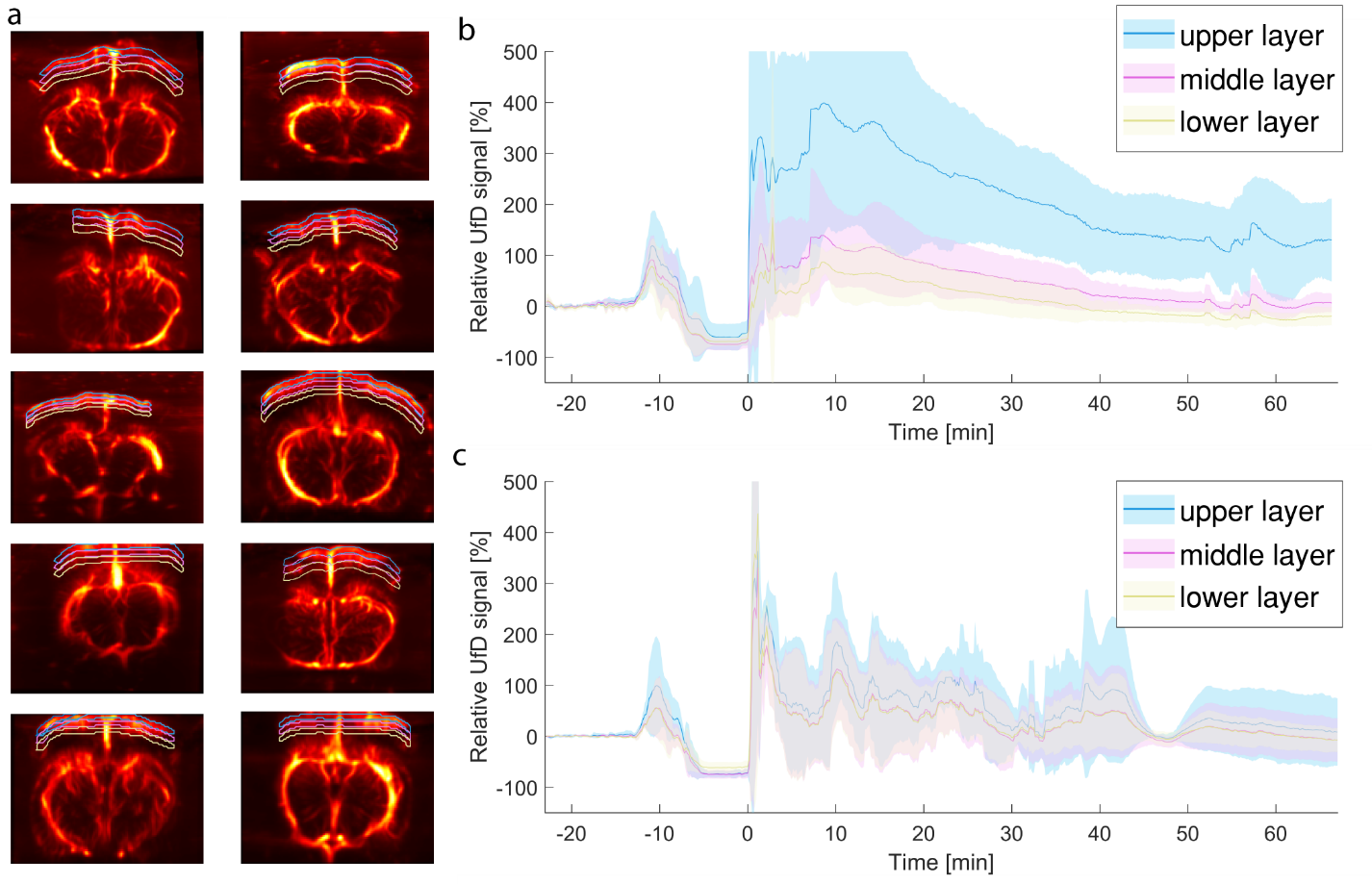


**Supplemental Figure 1**: 3-layer analysis of the cortical hyperemia in the cerebral cortex. **a**. The selection of the 3 layers is depicted for the 10 rabbits used in the study. **b**. Control case. Overall a strong hyperemia is observed in each layer of the cortex, but the hyperemia is much stronger near the surface of the cortex, with relative UfD variations reaching +400%. **c**. This difference between layers is mitigated in the case of the TLV group.
